# The Usefulness of the Forced Oscillation Technique in the Diagnosis of Bronchial Asthma in Children

**DOI:** 10.1155/2018/7519592

**Published:** 2018-07-24

**Authors:** L. Starczewska-Dymek, A. Bozek, M. Jakalski

**Affiliations:** ^1^Medical Center, Strzelce Opolskie, Poland; ^2^Clinical Department of Internal Disease, Dermatology and Allergology in Zabrze, Medical University of Silesia, Katowice, Poland

## Abstract

The forced oscillation technique (FOT) requires minimal patient cooperation and is useful for children. However, comprehensive values of respiratory impedance at baseline and after the reversibility test have not been definitively confirmed. The aim of this study was to evaluate the basic parameters of FOT reactance (Xrs) and resistance (Rrs) in groups of healthy children and children with controlled and uncontrolled asthma. The second aim was the assessment of the reversibility bronchial test using the forced oscillation method in children with bronchial asthma. *Materials and Methods*. One hundred and six children aged from 2 to 6 years diagnosed with early-onset controlled or uncontrolled asthma and healthy controls were included in this single-center, prospective, randomized study. All asthmatic patients and healthy controls underwent basic FOT as one measurement according to the recommendation of Resmon Pro FOT. The reversibility test was then performed 15 min after the administration of 200 mcg salbutamol by MDI in all patients. *Results*. Basic Rrs showed significantly higher mean values in patients with uncontrolled asthma compared to patients with controlled asthma, which were in turn higher than the values for patients in the control group (11.14 ± 1.29 versus 15.71 ± 2.6 versus 9.21 ± 0.98, resp.; *p* < 0.05). The data analysis showed similar relationships in terms of the Xrs between the studied groups (−4.76 ± 1.19 versus −7.31 ± 2.33 versus −2.11 ± 0.48, resp.; *p* < 0.05). According to the changes in the Rrs values, 35 (66%) positive bronchial reversibility tests were obtained in 53 subjects with controlled asthma and in 39 (74%) subjects with uncontrolled asthma. *Conclusions*. Rrs and Xrs obtained by FOT well-discriminate patients with asthma from healthy children. A bronchial reversibility test involving the use of FOT is valuable for the diagnosis of bronchial asthma.

## 1. Introduction

The forced oscillation technique (FOT) is a modern tool to estimate respiratory mechanics, but its clinical utility has not yet been established. The FOT is noninvasive and easy to use and demands minimal cooperation by patients to investigate pulmonary system mechanics [1]. This method is especially appropriate for patients who cooperate poorly during spirometry tests. Some data are available showing that this method could be useful for children [2–5]. Assessing airway obstruction is very important in the routine diagnosis of asthmatic children or children with wheezing syndrome [2, 6]. Asthma is characterized by reversible bronchoconstriction, and FOT also allows for the performance of the reversibility bronchial test, which is frequently impossible to perform in children due to ineffective cooperation to meet ATS/ERS criteria [2].

Respiratory resistance (Rrs) and reactance (Xrs) are the basic parameters that are assessed during inspiration and expiration in the breathing cycle. These two parameters describe the status of bronchial flows during measurements. Rrs is very sensitive to the degree of obstruction of the main central airways, while small airways (<2 mm in diameter) account for only 10% of total airway resistance. Resistance in healthy adult subjects is almost independent of the frequency of the stimulating pressure and becomes abnormally higher at lower frequencies during severe obstruction or positive bronchial challenge. Xrs is a measure of the elastic recoil forces of the respiratory system. This parameter enables clinicians to determine how effectively the lung is being ventilated in its distal areas or how well air reaches peripheral areas. Xrs falls below predicted values at low oscillating frequencies under the following conditions: peripheral obstruction, tidal expiratory flow limitation, alveolar gas trapping, and/or the closing of alveolar units [7].

The differences in the volume of Rrs and Xrs between baseline measurement and after salbutamol administration are useful for bronchial reversibility tests [4].

Despite the evidence that FOT is useful, it remains unappreciated in clinical practice. There is a lack of established baseline values according to age in healthy patients, and few studies have explored the usefulness of FOT in a group of children. It is important to determine whether FOT is able to discriminate asthmatic patients from healthy controls.

The primary goals of this study were to evaluate the basic parameters of FOT Xrs and Rrs in a group of healthy children and in children with controlled and uncontrolled asthma in terms of their clinical utility for discriminating children with controlled and uncontrolled asthma and healthy controls.

In addition, the utility of the reversibility bronchial test using the forced oscillation method in children with bronchial asthma was explored.

The secondary aim was to assess the influence of height, body weight, and atopic features on the values of reactance and resistance.

Achieving these goals might show the clinical usefulness of FOT as a diagnostic tool in asthmatic children.

## 2. Materials and Methods

### 2.1. Study Subjects

Patients diagnosed with asthma and a healthy control group were included in the study in an outpatient medical center in Poland. Patients who met the inclusion criteria (see below) were screened and randomized, and 106 children from 2 to 6 years of age diagnosed with early-onset asthma were selected from all 351 prescreened participants. Following the assessment of disease control, this group was divided into two subgroups: fully controlled asthma (53 children) and uncontrolled asthma (53 children). The control group included 45 healthy children who were randomly selected from 155 prescreened patients.

The criteria for inclusion in the study group were as follows:

1. Informed consent was provided by parents or legal guardians and patients (above 5 years of age) and was signed and dated.
2. Children aged 2 to 6 years diagnosed with chronic bronchial asthma with a minimum of a 3-month history of the diagnosis. Diagnosis of bronchial asthma based on the Martinez criteria and clinical history (including an assessment of the Asthma Predictive Index = API), physical examination, differential diagnosis of bronchial obstruction, and response to the included anti-inflammatory treatment. For children who were “early wheezers during the first 3 years of life,” API negative predictive values ranged from 93.9% at 6 years of age to 86.5% at 13 years of age. For children who were “early frequent wheezers during the first 3 years of life,” the negative predictive values were 91.6% and 84.2% for 6 and 13 years of age, respectively.
3. Bronchial asthma during anti-inflammatory treatment (inhaled glucocorticosteroids or inhaled glucocorticosteroids + montelukast + and a short beta agonist) for a minimum of 3 months.

### 2.2. Exclusion Criteria

Patients with a current, active infection of the respiratory system, infection of the lower respiratory tract 4 weeks prior to the study, chronic diseases other than bronchial asthma, prematurity aspiration of a foreign body in the airways in their history, and pulmonary inflammation or bronchiolitis during the first year of life were excluded from the study.

The control group contained healthy patients in the same age range. The criteria for inclusion in the control group were as follows:

1. Informed consents by parents or legal guardians and patients (greater than 5 years of age) were also signed and dated.
2. Children aged 2 to 6 years without a medical history or clinical evidence of bronchial asthma.

The exclusion criteria were the same as those for the study group.

### 2.3. Study Design

The study design was a prospective, randomized study. The study was approved by the Local Bioethical Committee in Opole, Poland. Medical history taking, diagnosis of asthma, and physical examination were performed before lung function assessments.

Participants including all asthmatic patients and healthy controls underwent one basic FOT measurement according to the recommendation of Resmon Pro FOT [8]. The reversibility test was then performed after the administration of salbutamol in all subjects. The postsalbutamol FOT was performed after 15 minutes.

### 2.4. Assessment of Bronchial Asthma

The assessment of controlled asthma in 2- and 3-year-olds included a questionnaire regarding the children's symptoms based on the recommendations of GINA experts (no validated tools are available to assess asthma control in children under 4 years). The questionnaire contained four questions about the occurrence of asthma symptoms in the last 4 weeks [9].

For the assessment of controlled asthma in children from 4 to 6 years of age, the C-ACT (childhood asthma control test) asthma test was carried out by the caregivers and children; this test included seven questions about the child's asthma symptoms during the past 4 weeks [9]. In the uncontrolled asthma group, patients with a sum of C-ACT points of 19 or less (for children 4 years and older) and with a GINA expert questionnaire score of 2 or more for children under 4 qualified for inclusion.

Patients with a C-ACT score of 20 or higher (children of 4 years and older) and those with a GINA questionnaire score below 2 points (children under the age of 4) were included in the controlled asthma group.

### 2.5. History of Atopic Diseases

Atopy was defined as the presence of allergic rhinitis and/or atopic dermatitis in the participants. A similar criterion was used to recognize atopy in the parents. Additionally, the results of allergen-specific IgE in the serum (Polycheck, Biocheck, Germany) for a total of 30 inhalation and food allergens were analyzed. A positive IgE concentration >0.35 kU/l was considered positive.

### 2.6. Physical Examination

During the visit, a general physical examination was carried out, including a rhinoscopy examination and assessments of height and weight.

### 2.7. Forced Oscillation Test (FOT)

The study was performed using the Resmon Pro device (Restech SRL, Italy; marketed by MGCD Diagnostic USA) in accordance with the guidelines and recommendations of the American Thoracic Society/European Respiratory Society (ATS/ERS) [2]. The device was calibrated every day. On the day of the study, the patients were asked not to use bronchodilators. The study was carried out using a single wave frequency of 8 Hz, as suggested for children. The standards were adopted for the Caucasian race [4]. The test was carried out in a sitting position with the head in a neutral position or with a slightly raised chin. Caregivers were asked to hold the child's cheeks with both hands. A clip was put on the nose. The children were asked to cover the disposable mouthpiece with their mouth so that the air could not escape and to breathe slowly and steadily. The software analyzed the data recorded during a minimum of 10 calm breathing cycles. For data analysis, the parameters of the total Rrs value and inspiratory and expiratory components, as well as the total Xrs and its inspiratory and expiratory components, were used. The validity of the measurement at 8 Hz was assessed automatically by the Resmon Pro software using the coherence function, which describes the statistical relationship between input and output signals. A coherence function >0.95 is considered an acceptable limit for Rrs and Xrs measurements. The measurements with a coherence function <0.95 were excluded automatically. Therefore, at least three acceptable measurements were analyzed. All studied patients achieved that level of coherence.

### 2.8. Reversibility of the Bronchial Obstruction Test

After performing the initial FOT study, all participants inhaled the short-acting beta 2-agonist salbutamol MDI 200 mcg through an inhalation chamber (Aerochamber Plus, Trudell Medical International, London, ON, Canada). After 15 minutes of taking the drug, the FOT examination was performed again.

The values of Rrs and Xrs were evaluated, including their inspiratory and expiratory components, before and after the administration of the bronchodilator.

For children, a bronchial reversibility test can be considered positive if the pre-postreduction on the total Rrs values measured at 8 Hz are >32% or 2.79 cm H_2_O/(L/s) [10].

### 2.9. Statistical Analysis

Statistica 8.2 computer program (SaftPol, Kraków) was used to analyze the results. Some results are presented as the mean and standard deviation or with 95% confidence intervals. The analysis used Student's *t* tests for related and unrelated samples and used chi-square and ANOVA tests for parameters that were not normally distributed. ROC curves were drawn to determine the cutoff value corresponding to the best compromise between sensitivity and specificity (defined as the point of the curve closest to the upper left-hand corner). Statistical significance was assumed for *p* < 0.05.

## 3. Results

The characteristics of the groups are presented in Table 1.

### 3.1. Analysis of Resistance and Reactance in the Studied Groups

#### 3.1.1. Rrs Analysis

Baseline mean Rrs was significantly higher in patients with uncontrolled asthma compared to patients with controlled asthma and in relation to the control group (healthy), as shown in Table 2.

#### 3.1.2. Xrs Analysis

The data analysis showed similar relationships to those in the case of resistance between the groups studied. The results are shown in Table 3.

### 3.2. Difference between the Inspiration and Expiration Components of Rrs and Xrs

In patients with controlled and uncontrolled asthma, significantly larger differences between inspiratory and expiratory Rrs were observed than those in healthy controls. No similar significant differences were observed during the Xrs analysis (Table 3).

### 3.3. Sensitivity and Specificity of FOT Parameters

**Figure 1:**
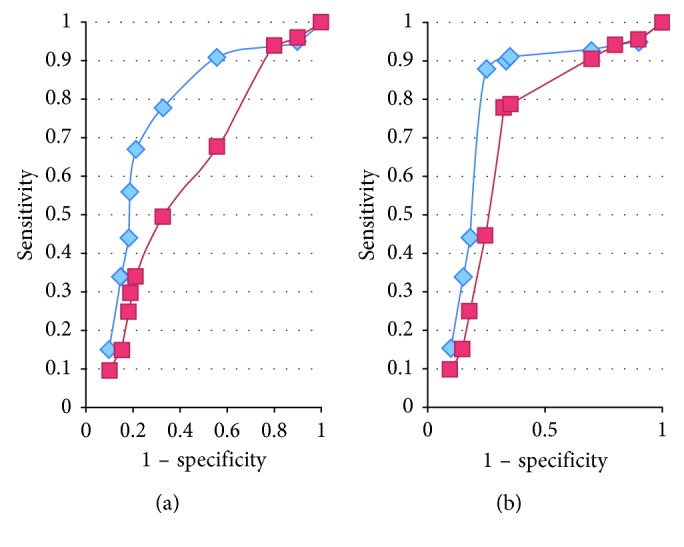
ROC curves corresponding to the sensitivity and specificity of possible cutoff points for Δ Rrs (diamonds) and Δ Xrs (squares) for discriminating children with asthma (control and uncontrol) and without it (a) and for discriminating children with controlled and uncontrolled asthma (b).

ROC curves corresponding to the sensitivity and specificity of possible cutoff points for Δ Rrs and Δ Xrs for discriminating between children with or without asthma (Figure 1(a)). A decrease in Δ Rrs of 1 yielded 74% sensitivity and 81% specificity and an increase in Δ Xrs of 1 yielded approximately 68% sensitivity and 64% specificity (Figure 1(b)). Similar discrimination between children with controlled and uncontrolled asthma was found: Δ Rrs yielded 83% sensitivity and 92% specificity, and Δ Xrs yielded 78% sensitivity and 89% specificity.

### 3.4. Evaluation of the Bronchial Reversibility Tests based on the Analysis of Resistance and Reactance in the Studied Groups

#### 3.4.1. Response to Salbutamol

In the group of patients with controlled asthma, a significant decrease in the mean total Rrs value was observed from 11.14 ± 1.29 to 6.51 ± 1.17 after salbutamol administration (*p*=0.001), and an increase in total Xrs was observed from −4.76 ± 1.19 to −1.15 ± 0.59 after salbutamol administration (*p*=0.003). According to the Calogero criteria, 35 (66%) positive tests were obtained in 53 subjects.

In patients with uncontrolled asthma, the following Rrs and Xrs values were obtained before and after salbutamol administration:

  Rrs: 15.71 ± 2.6 versus 10.29 ± 1.62 after salbutamol administration, *p*=0.001
  Xrs: −7.31 ± 2.33 versus −3.01 ± 1.21 after salbutamol administration, *p*=0.006

Analyzing the Rrs parameters, 39 (74%) positive tests were obtained in 53 subjects.

In the case of the control group, no significant changes were observed in the ranges of Xrs and Rrs after salbutamol administration:

  Rrs: 9.21 ± 0.98 versus 7.94 ± 2.09 after salbutamol administration, *p*=0.08
  Xrs: −2.11 ± 0.48 versus −2.07 ± 1.12 after salbutamol administration, *p*=0.12

One (2%) positive test was obtained in 45 subjects.

**Figure 2:**
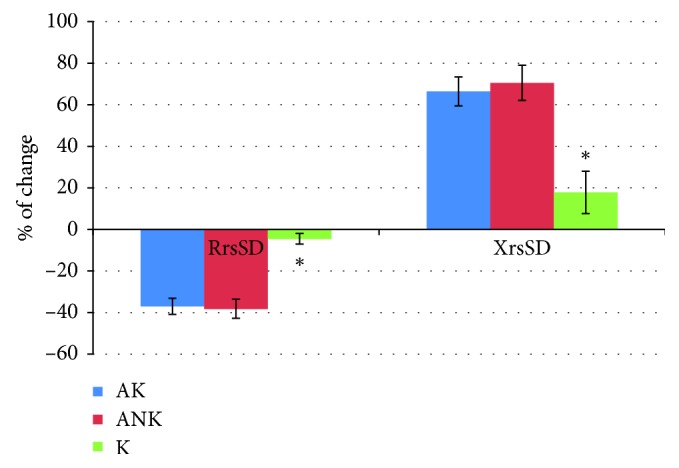
Percentage of changes in the Rrs and Xrs after salbutamol in comparison to baseline in the studied groups. XrsSD: average reactance with standard deviation; RrsSD: average resistance with standard deviation; ^*∗*^significant differences (ANOVA test) for *p* < 0.05; AK: patients with controlled asthma; ANK: patients with uncontrolled asthma; K: control group.

Differences in the analyzed parameters after the bronchial reversibility test are shown in Figure 2.

**Figure 3:**
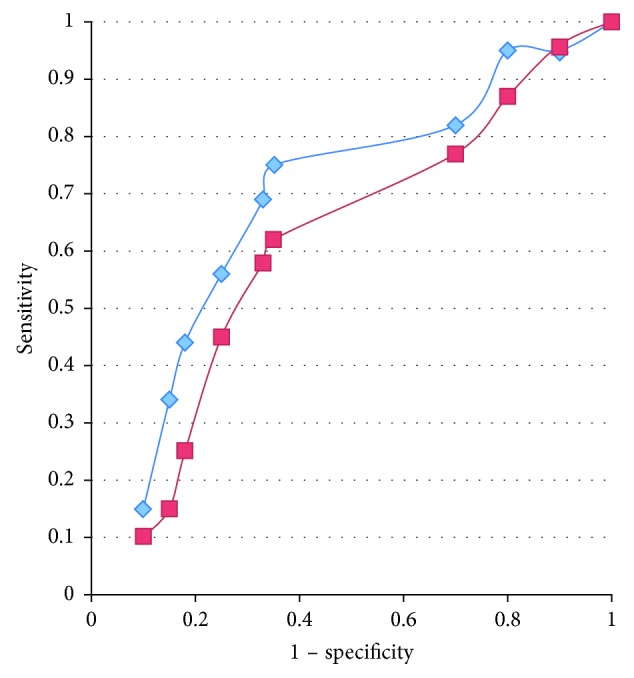
ROC curves corresponding to the sensitivity and specificity of possible cutoff points for changes in Rrs (diamonds) and Xrs (squares) pre- and postsalbutamol for discriminating children with asthma (control and uncontrol) and without it.

ROC curves corresponding to the sensitivity and specificity of possible cutoff points for changes between pre- and postsalbutamol Rrs and Xrs to discriminate between children with asthma (controlled and uncontrolled) and without asthma are shown in Figure 3.

A decrease in Rrs of 1 or more yielded 68% sensitivity and 75% specificity. An increase in Xrs of 0.6 yielded 57% sensitivity and 63% specificity.

The point of the ROC curve closest to the upper left-hand corner corresponded to a 29% decrease in Rrs after bronchodilator use and yielded 81% sensitivity and 69% specificity.

### 3.5. Height, Body Weight, Atopy, and FOT Parameters

**Figure 4:**
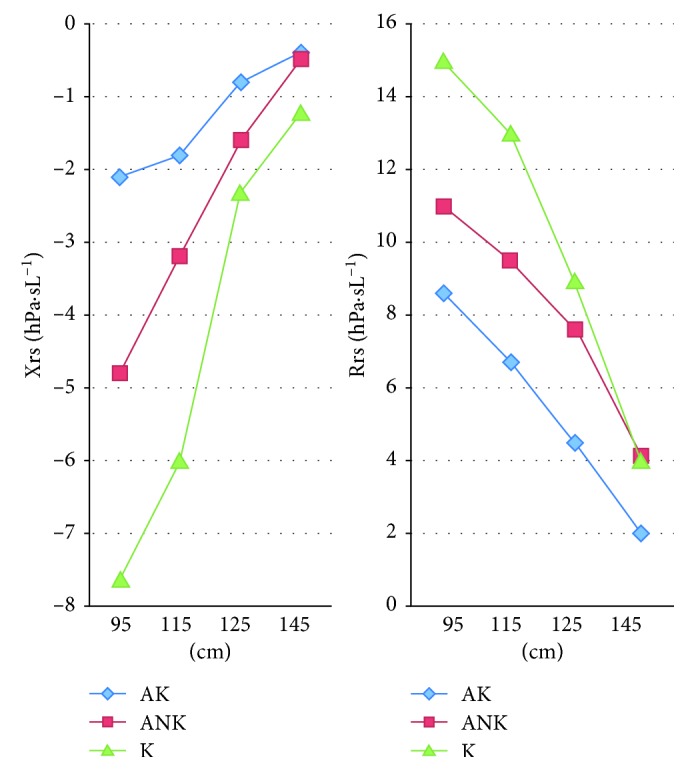
Xrs and Rrs in relation to height. AK: patients with controlled asthma; ANK: patients with uncontrolled asthma; K: control group.

The Rrs value decreased with patient height regardless of asthma, and Xrs increased in this case (Figure 4).

There was no relationship between weight and FOT parameters (Student's *t*-test, *p* > 0.05).

The presence of atopic features in the patient or in his family did not affect Rrs and Xrs values, and there were also no relationships between individual allergens and the number of positive test results and FOT parameters (chi-square test, *p* < 0.05). No dependence was seen for the occurrence of atopic dermatitis, allergic rhinoconjunctivitis, and food allergy (chi-square test, *p* < 0.05).

## 4. Discussion

The obtained results show that the measurement of FOT can discriminate between patients with asthma and healthy controls. Moreover, there were significant differences in the baseline values of Rrs and Xrs between patients with controlled and uncontrolled asthma. These results could be used to confirm that FOT is a useful tool in the diagnosis of asthma even without a reversibility test in children. However, there are various opinions about the utility of FOT in such cases. Some studies did not show abnormal resistance and reactance in asthmatic children [2, 11–13]. Large discrepancies in the results are explained by the different criteria used for including children with asthma, their different ages, and different starting values. Vu et al. emphasized that differences in Rrs and Xrs between asthmatics and control subjects were likely amplified by the timing of the measurement, which was performed soon after asthma exacerbation [3]. In the present study, these significant differences were obtained in patients without a present acute exacerbation. In addition to uncontrolled asthma, differences were apparent between controlled and stable asthma.

The discrepancy between the results was also influenced by the race of the patients examined. Data on patients studied by Lall et al. [14] and Vu et al. [3] are difficult to compare because of this feature.

The basic Rrs and Xrs values in healthy subjects were comparable to the patients in the study by Calagero et al., who examined healthy Italian preschool children [4, 10].

The differences between Rrs and Xrs might be dependent on the frequencies that were used. In our studies, 8 Hz was used because this value has been recommended by other authors [10]. According to the ATS/ERS criteria, all measurements should be rejected when the coherence is <0.95. This restriction improves the quality and reliability of the results.

Differences between the values of the inspiration and expiration components of Rrs and Xrs are important. The significant differences seen for the expiration and inspiration Rrs in asthmatic patients might explain the most important role of this parameter in the estimation of the mechanism of bronchoconstriction. This finding is consistent with other observations [4].

Several studies compared FOT with spirometry and/or whole body plethysmography for the assessment of airflow obstruction. Rrs measured by FOT and body plethysmography (sRaw) showed good correlation, but relationships between FOT and FEV_1_ were found. Some authors suggest that resistance measured by FOT, especially at low frequency, is a highly reproducible index and is compatible with FEV_1_; moreover, Rrs is a more sensitive parameter than FEV_1_ for the detection of airway obstruction [15].

The bronchodilator response as measured by the use of FOT is also valuable for assessing patients with asthma [16]. Only 2% of healthy controls had positive results; this outcome is a positive predictor of the specificity of this method.

The positive results (66 and 74%) for all asthmatics are evidence of the lower sensitivity of FOT. However, taking into account the young age of the population and the determined strict criteria of the positive test, the obtained results can be considered valuable. It is worth remembering that a reversibility test using spirometry is frequently impossible to perform for children, and the final results have limited specificity and sensitivity. Our data are consistent with those based on an Italian population, which observed changes in Rrs and Xrs following bronchodilator use that were related to the magnitude of the baseline values [10].

We also agree with Vu et al. that Rrs after salbutamol use would appear to be the most relevant parameter for diagnosing asthma; however, the improvement of Xrs after salbutamol administration might indicate a reversal of airflow limitation during tidal breathing [3].

A significant role of Rrs might be demonstrated by its significant change depending on the duration of asthma. There was no such observation for Xrs.

Relationships between height and FOT parameters were confirmed as there were consequences involving the enlargement and development of the airways. Standards were a decisive factor, as in spirometry. Atopy and weight did not influence the obtained results. This finding is consistent with the observations of other authors [3, 4].

Our study has some limitations, including the low number of patients, which does not allow us to draw conclusions about standards or to better discriminate patients with asthma from controls. We only focused on FOT, and there were no accompanying spirometry tests that could be compared. Such attempts were made, but due to weak cooperation, it was not possible to use this method in a further analysis. However, the results are proof that FOT is advantageous in such diagnostics.

## 5. Conclusion

FOT appears to be an important tool for the assessment of asthma in children. Basic parameters such as Rsr and Xrs are good factors for discriminating patients with asthma from healthy children. A bronchial reversibility test involving the use of FOT is valuable for the diagnosis of asthma, although its sensitivity can be limited. Further studies are needed.

## Figures and Tables

**Table 1:** Characteristics of the studied groups.

Feature	AK (*n*=53)	ANK (*n*=53)	K (*n*=45)	*P* ^*∗*^
Age	4.2 ± 1.3	4.8 ± 2.1	3.9 ± 1.2	NS
Women (%)	52.6	48.9	56.1	NS
Height (cm)	105.8 ± 8.9	110.1 ± 10.2	102.8 ± 12.1	NS
BMI	15.21	13.96	16.21	NS
Atopy (%)	26 (49.1)	27 (50.9)	24 (46.6)	NS
Time from the diagnosis of asthma in months (%)	19.5 ± 2.8	17.4 ± 4.2	—	NS
Atopy in family (%)	36 (67.9)	35 (66)	29 (64.4)	NS
AR (%)	25 (47.2)	21 (39.6)	20 (44.4)	NS
AD (%)	24 (45.3)	26 (49.1)	21 (46.7)	NS
Food allergy (%)	16 (30.2)	11 (20.8)	12 (26.7)	NS

AK: patients with controlled asthma; ANK: patients with uncontrolled asthma; K: control group; AR: allergic rhinitis; AD: dermatitis allergica; ^*∗*^Student's *t*-tests for related samples or the chi-square test were used.

**Table 2:** Comparison of Rrs values in the studied groups.

Rrs ( hPa·sL^−1^) Rrs (% of norm)	AK	ANK	K
Total	11.14^*∗*^	15.71^*∗∗*^	9.21^
	(95% CI: 9.93–13.17)	(95% CI: 13.49–18.11)	(95% CI: 7.59–9.86)

Inhaled	10.2^*∗*^	13.82^*∗∗*^	8.21^
	(95% CI: 8.53–11.95)	(95% CI: 12.03–14.05)	(95% CI: 6.23–8.84)

Exhaled	13.21^*∗*^	16.81^*∗∗*^	9.89^
	(95% CI: 11.13–14.82)	(95% CI: 15.11–17.03)	(95% CI: 7.64–11.15)

Δ Rrs	0.68^*∗*^	0.87^*∗∗*^	0.36^
	(95% CI: 0.61–0.73)	(95% CI: 0.75–1.02)	(95% CI: 0.26–0.47)

AK: patients with controlled asthma; ANK: patients with uncontrolled asthma; K: control group; Δ Rrs: the difference between the inspiration and expiration component for the resistance; significant differences (ANOVA test) between the following: ^*∗*^AK and K, *p* < 0.05; ^*∗∗*^ANC and K, *p* < 0.05; ^AK and ANC, *p* < 0.05.

**Table 3:** Comparison of Xrs values in the studied groups.

X_rs_ (hPa sL^−1^) X_rs_ (% norm)	AK	ANK	K
Total	−4.76^*∗*^	−7.31^*∗∗*^	−2.11^
	(95% CI: −5.21; −3.32)	(95% CI: −8.21; −5.54)	(95% CI: −3.09; −1.12)

Inhaled	−4.34^*∗*^	−6.71^*∗∗*^	−1.84^
	(95% CI: −5.68; −3.12)	(95% CI: −7.1; −5.89)	(95% CI: −2.81; −0.82)

Exhaled	−5.76^*∗*^	−8.49^*∗∗*^	−2.89^
	(95% CI: −7.02; −4.12)	(95% CI: −9.91; −7.52)	(95% CI: −3.81; −1.19)

Δ Xrs	0.42	0.46	0.39
	(95% CI: 0.36–0.51)	(95% CI: 0.41–0.62)	(95% CI: 0.29–0.47)

AK: patients with controlled asthma; ANK: patients with uncontrolled asthma; K: control group; Δ Xrs: the difference between the inspiration and expiration component of the reactance; significant differences (ANOVA test) between the following: ^*∗*^AK and K, *p* < 0.05; ^*∗∗*^ANC and K, *p* < 0.05; ^AK and ANC, *p* < 0.05.

## Data Availability

The data used to support the findings of this study are available from the corresponding author upon request.
